# A virtual ward for home hemodialysis patients – a pilot trial

**DOI:** 10.1186/s40697-015-0072-7

**Published:** 2015-11-01

**Authors:** Michael J. Raphael, Annie-Claire Nadeau-Fredette, Karthik K. Tennankore, Christopher T. Chan

**Affiliations:** Division of Nephrology, Toronto General Hospital - University Health Network, University of Toronto, Toronto, ON Canada; Division of Nephrology, Hôpital Maisonneuve-Rosemont, Université de Montreal, Montreal, QC Canada; Division of Nephrology, Dalhousie University/Nova Scotia Health, Halifax, NS Canada

**Keywords:** Care gaps, Emergency department visits, Home hemodialysis, Readmissions, Virtual ward

## Abstract

**Background:**

Patients with end-stage renal disease (ESRD) have a high rate of hospitalization and are prone to care gaps that may occur during the transition from hospital to home. The virtual ward (VW) is an innovative model that provides short-term transitional care to patients upon hospital discharge. The VW may be an effective intervention to address care gaps.

**Objectives:**

The primary objective of the pilot study was to assess the feasibility and practicality of implementing the Home Dialysis VW (HDVW) on a broader scale.

**Design:**

The HDVW Pilot Study enrolled home hemodialysis patients following one of four inclusion criteria: 1. Discharge from hospital, 2. Completion of an in-hospital medical procedure, 3. Prescription of an antibiotic, 4. Completion of home hemodialysis training. Patients were followed in the HDVW for 14 days and during this time were assessed serially with a clinician-led telephone interview for one of three transitional care gaps: 1. Requirement for change in hemodialysis prescription, 2. Requirement for coordination of follow-up care, 3. Requirement for medication change.

**Setting:**

The study was conducted in Toronto, Ontario, Canada at a quaternary care academic teaching hospital from 2012–2013.

**Patients:**

This study included 52 HDVW admissions among 35 patients selected from the existing home hemodialysis program.

**Measurements:**

The primary outcome was the identification of the number of care gaps at each HDVW admission. Secondary outcomes included the identification of potential predictors of care gaps and description of clinical adverse events following HDVW admission (readmissions, emergency department visits, unplanned visits to the home hemodialysis in-center).

**Results:**

The implementation and execution of the HDVW Pilot Study proved to be technically feasible and practical. A care gap was identified in 35 (67 %) of the HDVW admissions. In total, the cohort experienced 85 care gaps. There were no baseline demographic characteristics predictive of experiencing a care gap. In the total cohort observed for 2912 patient days, there were 9 readmissions, 13 visits to the emergency department, and 7 unplanned visits to the home hemodialysis in-center unit.

**Limitations:**

The results of this study are limited by the small study size and single-center experience.

**Conclusion:**

The implementation of a virtual ward for home hemodialysis patients is practical, feasible and identifies many care gaps which have the potential to result in subsequent adverse events. A larger, multi-center prospective clinical trial is justified to identify if the HDVW can prevent adverse events among home dialysis patients.

## Abrégé

**Informations connues:**

Les patients atteints d’insuffisance rénale terminale (IRT) sont hospitalisés fréquemment et sont particulièrement exposés aux lacunes de soins durant la période de transition entre leur hospitalisation et leur retour à la maison. L'unité virtuelle (UV) est un modèle innovateur de suivi qui permet de prodiguer des soins de transition à court terme pour les patients atteints d’IRT à leur sortie de l’hôpital. L’UV pourrait s’avérer un moyen efficace d’aborder et de combler les lacunes dans les soins et la prise en charge des patients lors de cette période de transition.

**Objectifs de l’étude:**

L’objectif principal de l’étude pilote était de vérifier la faisabilité et l’aspect pratique de la mise en œuvre d'une UV de dialyse à domicile à grande échelle .

**Plan de l’étude:**

Le recrutement des participants pour l’étude pilote s’est fait parmi les patients pratiquant déjà l’hémodialyse à domicile, et selon l’un ou l’autre des critères suivants :

1. Le patient devait avoir reçu son congé de l’hôpital.
2. Le patient devait avoir complété une procédure ou intervention intra-hospitalière.
3. Le patient devait suivre un traitement aux antibiotiques.
4. Le patient devait avoir complété une formation de base pour pratiquer l’hémodialyse à domicile.

Ces patients ont été suivis via UV sur une période de quatorze jours au cours desquels ils devaient se soumettre à des interviews téléphoniques effectués par un membre du personnel clinique qui était chargé d’évaluer l’une ou l’autre de ces fréquentes lacunes dans les soins transitoires :

1. La nécessité de réviser l’ordonnance d’hémodialyse
2. Le manque de coordination dans l’offre de soins en suivi post-hospitalisation
3. La nécessité de rectifier la médication prescrite

**Contexte de l’étude:**

Cette étude a été menée entre 2012 et 2013 dans un hôpital d’enseignement des soins de santé quaternaires à Toronto (ON), Canada.

**Patients:**

Cette étude pilote porte sur 52 épisodes d'admission à l'UV de dialyse à domicile vécues par 35 patients recrutés au sein du programme d'hémodialyse à domicile déjà existant dans ce centre hospitalier.

**Mesures:**

L’objectif principal était de déterminer le nombre de lacunes rencontré par chaque patient admis à l'UV de dialyse à domicile . Les objectifs secondaires incluaient l’identification de caractéristiques permettant de prévoir les lacunes de soins auxquelles se heurteront les patients, et la description des événements cliniques indésirables pouvant survenir à la suite d’une admission à l'UV de dialyse à domicile , soit une réadmission aux unités de soins néphrologiques, une visite aux urgences ou une visite non planifiée à l'unité d'hémodialyse à domicile de l’hôpital.

**Résultats:**

La mise en œuvre de l'UV de dialyse à domicile s’avère réalisable du point de vue logistique, et utile pour les patients atteints d’IRT. L’étude pilote a pu identifier au moins une lacune de soins dans 35 (67 %) des admissions au service. En tout, la cohorte de patients admis au service a fait face à 85 lacunes de soins dans la période de transition entre l’hospitalisation et le retour à la maison. Aucune caractéristique démographique de base ne permettait de prévoir que des patients allaient éprouver des lacunes dans les soins. Durant un suivi total de 2912 jours-patients, 9 réadmissions aux unités de soins néphrologiques, 13 visites aux urgences et 7 visites non planifiées à l'unité d'hémodialyse à domicile de l'hôpital ont été enregistrées.

**Limites de l’étude:**

Les résultats sont limités par la taille réduite de l’étude et par le fait que celle-ci n’ait été menée que dans un seul centre hospitalier.

**Conclusions:**

La mise en œuvre de l'unité virtuelle (UV) pour les patients qui pratiquent l’hémodialyse à domicile s’avère techniquement réalisable et utile pour identifier les lacunes dans les soins qui peuvent éventuellement engendrer des événements cliniques indésirables. Afin de déterminer si un tel service permettrait de prévenir les événements cliniques indésirables chez les patients qui pratiquent la dialyse à domicile, il serait justifié de réaliser un essai clinique prospectif à plus grande échelle, au sein d’un plus grand nombre de centres hospitaliers.

## What was known before

Patients with end-stage renal disease (ESRD) have a high rate of hospitalization and are prone to care gaps that may occur during the transition from hospital to home. There are no existing comprehensive transitional care management plans designed specifically for patients with ESRD.

## What this study adds

Implementation of a virtual ward designed specifically for patients with end-stage renal disease is feasible, practical and identifies many transitional care gaps that have the potential to result in serious adverse events.

## Background

The Virtual Ward (VW) is an integrative, multidisciplinary case-management model that serves to provide short-term transitional care to patients upon discharge from hospital [1]. The VW model was originally designed in the United Kingdom in 2004 with the aim of preventing unplanned hospital admissions among high risk patients [2]. Key elements of the VW include the use of predictive modeling to identify patients at high-risk of unplanned hospitalization, multidisciplinary care delivered while the patient resides in their own home, and office-based patient management discussion rounds [1, 3].

The VW model is aptly suited for use in patients with end-stage renal disease (ESRD). These patients are medically complex, have sophisticated care needs and have a high rate of hospitalization and readmission [4–6]. Their rate of all-cause hospitalization is 37 % greater than the general population with an average of 1.73 hospital admissions per year [4]. Furthermore, the 30-day readmission rate is 35.2 % [4].

Related to their frequent hospitalizations, patients with ESRD are prone to care gaps that may occur during the transition from hospital to home [7]. During these transitional periods patients are at an increased risk of experiencing an adverse event (AE) because of changes to their health status, medication regimen and care plan that occur during hospitalization [8–11]. In the general medical population, half of patients experience at least one medical error upon discharge from hospital [12]. As a result, nearly a quarter of patients may suffer an AE [13].

Effective strategies to mitigate transitional care gaps in the ESRD population are not well described in the literature. To this end, we sought to leverage the existing infrastructure of our home hemodialysis program to develop a virtual ward for ESRD patients. We hypothesized that implementation of a HDVW would identify a significant number of care gaps that may be actionable to prevent AE. Here, we report the experience of the pilot trial.

## Methods

### Study population

After obtaining approval from the University Health Network Research Ethics Board, we prospectively enrolled 35 patients in the pilot phase of our HDVW to assess its feasibility and practically. The cut off sample size for the pilot study was selected by consensus from the authors when feasibility of the program was felt to be demonstrated. The sample size for the pilot study was not based on formal sample size calculations. All patients provided written informed consent for participation in the study. Patients were eligible to be included in the virtual ward if they were existing members of the home hemodialysis program and met any one of four inclusion criteria: 1. Discharged from hospital following an inpatient admission, 2. Completion of an in-hospital medical procedure (E.g. Interventional access procedure), 3. Received a prescription for an oral or intravenous antibiotic, 4. Completion of the home hemodialysis training program. Patients were excluded from enrollment in the HDVW if consent was declined or a language barrier precluded telephone follow-up.

### Objectives and outcomes

The primary objective of the pilot study was to assess the feasibility and practicality of implementing the HDVW on a broader scale [14]. The primary outcome was the number of care gaps observed. Care gaps were abstracted into three pre-defined categories: 1. Requirement for change in hemodialysis prescription, 2. Requirement for coordination of follow-up care, 3. Requirement for medication change. Secondary outcomes included the identification of potential predictors of care gaps and description of clinical adverse events within eight weeks of HDVW admission (readmissions, emergency department visits, unplanned visits to the home hemodialysis in-center). The index cohort of patients in the pilot study could be re-enrolled into the HDVW if they once again met inclusionary criteria. At the point of re-enrollment, they were censored from secondary outcome follow-up.

### Virtual ward intervention

The detailed design and development of the HDVW has been previously described in the literature [15]. Patients were followed in the HDVW for 14 days and during this time were assessed serially with a clinician-led telephone interview for the presence of any potential care gaps. By protocol, patients were telephoned once per week and administered a standardized patient assessment tool. At each assessment, a nephrologist, clinical nephrology trainee or home hemodialysis nurse would review current medications and recent laboratory investigations and document dialysis hemodynamics and body weight. The clinician would also document any patient specific concerns and the Modified Edmonton Symptom Assessment Scale (mESAS) [16]. At the end of each assessment, the physician or nurse would create an issues-based patient-specific problem list and provide education or care recommendations to the patient. All prescriptions for new medications and medical recommendations were reviewed with, and approved by, an attending nephrology staff. The study protocol mandated at least one call per week; however, if issues were identified that required more rapid follow-up patients would be called on an as needed basis until the issue was resolved.

### Statistical analysis

Baseline demographics were stratified by presence or absence of an identified care gap. Data are presented as counts with accompanying percentiles or medians with interquartile range, as appropriate. Differences between groups were analyzed by the Fisher’s exact test for categorical data and Mann–Whitney U test for continuous non-normally distributed data.

Potential predictors of care gaps were analyzed using unadjusted and adjusted multivariable logistic regression with robust standard error to account for clustered data. Variables included in the exploratory multivariable logistic regression were pre-specified based on biological plausibility and included age, Charlson Comorbidity Index (CCI) and duration of renal replacement therapy. The secondary outcomes are presented as descriptive data.

Statistical analysis was performed using the statistical software Stata IC (version 13.1 StataCorp, College Station, TX) [17]. All P values are two-sided with statistical significance set to *P* <0.05.

## Results

The implementation and execution of the HDVW Pilot Study proved to be technically feasible and practical. This study included 52 HDVW admissions among 35 patients. Thirty-five (67 %) of the 52 HDVW admissions had at least one identifiable care gap. The mean age of the entire cohort was 49.3 and the median CCI was 5. On average, patients had 14.0 years on renal replacement therapy and 3.8 years on home hemodialysis. The baseline characteristics of those with and without care gaps are presented in Table 1.

**Table 1:** Demographics of patients with and without care gaps

	No care gap	Care gap	*P*
Number of VW admissions	17	35	
Age	58 (40–61)	49 (41–55)	0.27
Male	8 (47)	17 (49)	1.00
Cause of end stage renal disease			0.19
Glomerulonephritis	7 (41)	5 (14)	
Diabetes	3 (18)	2 (6)	
Congenital disease	2 (12)	8 (23)	
Calcineurin inhibitor toxicity	2 (12)	8 (23)	
Hemolytic uremic syndrome	1 (6)	2 (6)	
Systemic lupus erythematous	0	3 (9)	
Renal cell carcinoma	0	2 (6)	
Hypertension	1 (6)	1 (3)	
Vasculitis	0	1 (3)	
Undetermined	1 (6)	3 (9)	
Duration of RRT (years)	8.8 (1.4–19.1)	12.2 (3.6–20)	0.35
Duration of HHD (years)	2.3 (0.6–4.8)	3.3 (0.7–5.5)	0.74
Charlson comorbidity Index (CCI)	4 (3–6)	5 (4–7)	0.22
Cancer history	6 (35)	12 (34)	1.00
Previous transplant	7 (41)	17 (49)	0.77
Permanent vascular access^a^	4 (25)	12 (35)	0.53
Admission criteria			0.07
Hospital discharge	14 (82)	30 (86)	
Completed medical procedure	2 (12)	0	
Antibiotic prescription	1 (6)	1 (3)	
Completed HHD post training	0	4 (11)	

Values presented as median (Interquartile range) and count (%). Percentages may exceed 100 due to rounding. VW = Virtual Ward, RRT = Renal Replacement Therapy, HHD = Home hemodialysis. ^a^Native arteriovenous fistula or graft

In total, the cohort experienced 85 care gaps including 25 changes in hemodialysis prescription, 35 gaps in coordination of follow-up care and 25 medication changes. A detailed breakdown of the categorized care gaps is presented in Table 2.

**Table 2:** Detailed description of addressed care gaps

Change in hemodialysis prescription		
	Alter target weight	17
	Alter frequency of dialysis	3
	Alter dialysis anticoagulation	3
	Alter dialysate bath	2
Coordination of follow-up care		
	Arrange follow-up testing	14
	Arrange follow-up appointment	14
	Dialysis technique education	6
	Other	3
	Home dialysis machine maintenance	1
Medication change		
	Analgesic	7
	Anti-hypertensive	3
	Non-dialysis anticoagulant	4
	Constipation medication	3
	Gastroesophageal reflux medication	2
	Iron supplementation	2
	Electrolyte medication	2
	Immunosuppressant	1
	Anti-pruritic	1

Values presented as counts

There were no baseline demographic characteristics predictive of experiencing a care gap (Table 3). In an exploratory multivariable logistic regression model, increasing age was associated with experiencing fewer care gaps (Odds ratio (OR) 0.93 per year, 95 % confidence interval (CI), 0.88–0.99; *P* = 0.01); while a higher CCI score was associated with experiencing more care gaps (OR per CCI score increase 1.60, 95 % CI, 1.12–2.29; *P* = 0.007). The fewer care gaps associated with increasing age could not be accounted for by a longer history of experience with renal replacement therapy (RRT) (Table 3).

**Table 3:** Unadjusted logistic regression and exploratory multivariable logistic regression for predictors of experiencing a care gap

	Unadjusted model			Multivariable adjusted model		
	OR	95 % CI	*P*	OR	95 % CI	*P*
Age (per year)	0.97	0.92–1.02	0.29	0.93	0.88–0.99	0.01
Age ≥60 years	0.36	0.11–1.13	0.08	–	–	–
Male	1.06	0.35–3.27	0.92	–	–	–
Primary kidney disease GN/autoimmune (vs. others)	0.75	0.24–2.27	0.61	–	–	–
Duration of RRT (per year)	1.02	0.98–1.06	0.26	1.01	0.98–1.05	0.47
Duration of HHD (per year)	1.03	0.89–1.21	0.67	–	–	–
CCI (per point)	1.24	0.97–1.59	0.08	1.60	1.12–2.29	0.007
Cancer history	0.96	0.21–2.85	0.94	–	–	–
Previous transplant	1.35	0.46–3.99	0.59	–	–	–
Permanent vascular access^a^ (vs. CVC)	1.64	0.48–5.60	0.43	–	–	–
Dependency status (vs. independent)	0.81	0.27–2.44	0.71	–	–	–
Reason for VW admission – hospital discharge (vs. others)	1.29	0.28–5.82	0.74	–	–	–

GN = Glomerulonephritis, RRT = Renal Replacement Therapy, HHD = Home hemodialysis, CVC = Central Venous Catheter, VW = Virtual Ward. ^a^Native arteriovenous fistula or graft, CCI = Charlson Comorbidity Index

Baseline mESAS were recorded in all but one enrollment into the HDVW. Unfortunately, 18 of 52 (35 %) exposures had incompletely documented HDVW discharge mESAS scores. This precluded meaningful analysis of the effect of the HDVW on patient symptom scores.

Analysis of the secondary outcomes in the total cohort observed for 2912 patient days identified 9 hospital readmissions, 13 visits to the emergency department, and 7 unplanned visits to the home hemodialysisin-center unit. There was no difference in any of the secondary outcomes when stratified by the presence or absence of a care gap (Table 4).

**Table 4:** Secondary outcomes stratified by presence of absence of care gaps

	No care gap	Care gap	*P*
Home hemodialysis unit visits			0.33
None	15 (88)	32 (91)	
1	2 (12)	1 (3)	
2	0	2 (6)	
Emergency visits			0.73
None	14 (82)	27 (77)	
1	2 (12)	7 (20)	
2	1 (6)	1 (3)	
In-Center hemodialysis sessions			1.00
None	17 (100)	33 (94)	
1	0	2 (5)	
Admissions			0.70
None	15 (88)	28 (80)	
1	2 (12)	7 (20)	

Values presented as counts and percentages

## Discussion

This study has demonstrated that creation of a virtual ward for home hemodialysis patients is technically feasible and practical. A large number of care gaps were identified which may result in adverse events including hospital readmission, morbidity and mortality. To our knowledge, this is the first report of a comprehensive, multimodal care plan targeted to improve the care of ESRD patients during the vulnerable transitional period following hospital discharge and after major changes in care.

We were unable to identify any significant differences in the baseline demographics of those who did and did not experience a care gap (Table 1). In a multivariable model, increasing age was associated with experiencing fewer care gaps. This association could not be accounted for by a longer duration of experience with RRT. This is seemingly congruent with the documented higher rate of re-hospitalization seen among younger hemodialysis patients in the United States Renal Data System (USRDS) reporting database [4]. In the USRDS database, the decreased rate of re-hospitalization among older patients with ESRD could not be fully attributed to the competing risk of increased mortality in older patients [4].

Dhalla et al. recently reported their experience in Toronto with a large, well-conducted, randomized-control trial comparing a VW strategy versus usual care in a diverse group of general medical patients at high-risk for readmission [18]. In their trial, despite an intensive VW intervention, no differences were found in rates of emergency department visits, readmission or death within 1 year of index hospital discharge. Accordingly, Dhalla et al. concluded that their VW model would not represent a cost-effective use of health care resources. In comparison, the HDVW focuses on a single patient population and benefits from being able to leverage the infrastructure of the pre-existing home-hemodialysis program which has been demonstrated to be 20-50 % less expensive than in-center hemodialysis [19]. The unique feature of a pre-existing infrastructure for home based patient care sets our model apart from any other transitional care intervention to date [20].

As evidenced by a rate of 30 day re-hospitalization that is double that of the general medical population, the current state of post-hospital discharge care for patients with ESRD remains suboptimal [4, 7, 21]. Interventions specifically designed to improve this period of patient vulnerability remain sparse. Recent population-level data has suggested that simple interventions may contribute to improved transitional care for patients with ESRD. Erickson et al. found that one additional clinician visit within 30 days following hospital discharge may reduce the 30 day readmission rate by 3.5 % [22]. Similarly, Chan et al. identified that evaluation and management of anemia and resumption of Vitamin D within 7 days of hospital discharge are associated with reduced re-hospitalization rates [23].

While these specific interventions may contribute to improving the post-hospitalization care of patients with ESRD, a comprehensive, multimodal care model is urgently needed. For this reason, we feel that evaluation of the HDVW with a larger, multi-center study is indicated. This prospective study will enable us to characterize better the care gaps experienced in the post-hospital transitional period and whether or not intervention can reduce the rate of adverse events including readmission. Furthermore, while it is well documented that home hemodialysis programs are able to reduce costs, it will be important to perform a costs analysis after implementation of the HDVW prospective trial [19]. Given that the HDVW does not require additional investment in resources beyond that to run the home-hemodialysis program, if the intervention proves effective in preventing emergency department visits and readmissions there is great potential for further cost saving.

Our study has several limitations and has highlighted several areas that will require attention prior to implementation of the HDVW on a larger, multicenter scale [15]. Consistent with the design of a pilot study, the small number of patients limits the assessment of the incidence of care gaps, emergency department and unplanned in-center visits and readmissions [14]. Likewise, it limits the statistical power to identify predictors of care gaps and unplanned hospital utilizations. This limitation might be overcome in a prospective multicenter study of a HDVW where we will also be able to collect more information on predictors of care gaps including duration and indication for index hospital admission. Furthermore, data recording by the individual clinician may have incompletely captured events, weakening the dataset. While a thorough baseline medical history, demographic assessment and baseline symptom assessment was documented in almost all patients; patient symptoms at discharge were not consistently recorded. Accordingly, no conclusions can be made regarding the impact of the HDVW on patient symptoms. However, this reinforces the importance of documenting the mESAS on discharge from the HDVW in the prospective multicenter trial.

## Conclusion

The implementation of a virtual ward for home hemodialysis patients is practical, feasible and identifies many care gaps which have the potential to result in subsequent adverse events. A larger, multi-center prospective clinical trial is justified to identify if the HDVW can prevent adverse events among home dialysis patients.
